# INFLUENCE OF EARTH, WIND, AND FIRE ON WELL-BEING: THE CLASSIC CONCENTRIC CIRCLES MODEL OF CONTEXT IS ALSO FROM THE 1970S

**DOI:** 10.1093/geroni/igac059.1444

**Published:** 2022-12-20

**Authors:** Michelle Ng, Angelina Garron, Denis Gerstorf, Nilam Ram

**Affiliations:** Stanford University, Palo Alto, California, United States; Stanford University, Palo Alto, California, United States; Humboldt Universität zu Berlin, Berlin, Berlin, Germany; Stanford University, Stanford, California, United States

## Abstract

Classic bioecological models of human development (Bronfenbrenner, 1979) highlight the influence that five layers of context have on individuals’ function and growth. Modern versions specifically highlight five specific aspects of context (socio-economic, social, physical, care/service, and technology) that influence both how older adults negotiate their daily lives and how they age. Using a collection of empirical findings obtained from our analyses of longitudinal panel data from the German SOEP, experience sampling data from iSAHIB, and screenome data from the Stanford HSP, we illustrate how differences in individuals’ engagement with green spaces (earth), ambient air pollution (wind), and smartphones (fire) may contribute to differences in daily emotional well-being and developmental changes in life satisfaction. We then use these examples to elucidate shortcomings of the hierarchical models used to frame investigations of context and development for the past 40 years – and suggest the classic models be renewed rather than continually recycled.

